# Multilocus Sequence Typing of Genital *Chlamydia trachomatis* in Norway Reveals Multiple New Sequence Types and a Large Genetic Diversity

**DOI:** 10.1371/journal.pone.0034452

**Published:** 2012-03-28

**Authors:** Kirsten Gravningen, Linus Christerson, Anne-Sofie Furberg, Gunnar Skov Simonsen, Kristina Ödman, Anna Ståhlsten, Björn Herrmann

**Affiliations:** 1 Department of Microbiology and Infection Control, University Hospital of North Norway, Tromsø, Norway; 2 Faculty of Health Sciences, University of Tromsø, Tromsø, Norway; 3 Section of Clinical Bacteriology, Department of Medical Sciences, Uppsala University, Uppsala, Sweden; University of California Merced, United States of America

## Abstract

**Background:**

The *Chlamydia trachomatis* incidence rate in Finnmark, the most northern and sparsely populated county in Norway, has been twice the national average. This population based cross-sectional study among Finnmark high school students had the following aims: i) to examine distribution of multilocus sequence types (STs) of *C. trachomatis* in a previously unmapped area, ii) to compare chlamydia genetic diversity in Finnmark with that of two urban regions, and iii) to compare discriminatory capacity of multilocus sequence typing (MLST) with conventional *ompA* sequencing in a large number of chlamydia specimens.

**Methodology:**

*ompA* sequencing and a high-resolution MLST system based on PCR amplification and DNA sequencing of five highly variable genetic regions were used. Eighty chlamydia specimens from adolescents aged 15–20 years in Finnmark were collected in five high schools (n = 60) and from routine clinical samples in the laboratory (n = 20). These were compared to routine clinical samples from adolescents in Tromsø (n = 80) and Trondheim (n = 88), capitals of North and Central Norway, respectively.

**Principal Findings:**

*ompA* sequencing detected 11 genotypes in 248 specimens from all three areas. MLST displayed 50 STs providing a five-fold higher resolution. Two-thirds of all STs were novel. The common *ompA* E/Bour genotype comprised 46% and resolved into 24 different STs. MLST identified the Swedish new variant of *C. trachomatis* not discriminated by *ompA* sequencing. Simpson's discriminatory index (*D*) was 0.93 for MLST, while a corrected *D_c_* was 0.97. There were no statistically significant differences in ST genetic diversity between geographic areas. Finnmark had an atypical genovar distribution with G being predominant. This was mainly due to expansion of specific STs of which the novel ST161 was unique for Finnmark.

**Conclusions/Significance:**

MLST revealed multiple new STs and a larger genetic diversity in comparison to *ompA* sequencing and proved to be a useful tool in molecular epidemiology of chlamydia infections.

## Introduction

Despite widespread efforts to control *Chlamydia trachomatis*, it remains the leading cause of bacterial sexually transmitted infections in Scandinavia and worldwide. The prevalence is highest among 15–24 year-olds [1]. In Norway, genital chlamydia infections have been part of the national surveillance system for communicable diseases since 2003. Treatment is free of charge, and partner tracing is compulsory. As in other western countries having implemented extensive chlamydia testing, the reported number of chlamydia infections in Norway almost doubled since the mid-1990s [1]. The highest incidence rates have been reported in Finnmark, the most northern and sparsely populated county in Norway with an incidence rate of 8.98/1000 in 2009, almost twice the national average (4.67/1000) [2].

Strain typing of *C. trachomatis* is important to understand the genetic population structure and is a useful tool in epidemiological studies, in investigation of infection transmission or recurrence, in sexual network analysis, and in surveillance of emerging strains such as the Swedish new variant of *C. trachomatis* (nvCT) [3]. It is assumed that persons infected by the same chlamydia strain are more likely to be epidemiologically linked than those infected with different strains. Traditional typing differentiated genital *C. trachomatis* into subgroups based on serospecificity for the major outer membrane protein (MOMP), encoded by the *ompA* gene. MOMP and *ompA* based methods have predominated typing in the past decades [4] where sequencing of the *ompA* gene has provided the best discriminatory capacity [5]. As these methods identified only a limited number of distinct subtypes, and the various subtypes could persist for a long time within a geographic area, research has focused on developing strain typing techniques with higher capacity of resolution. Several alternative typing systems for *C. trachomatis* have been published in recent years. Two standard multilocus sequence typing (MLST) approaches based on housekeeping genes have a discriminatory capacity comparable to *ompA* and could be useful for slowly evolving processes in evolutionary studies, but were not used in this study due to limited resolution [6], [7]. A significantly higher resolution has been shown for a multilocus variable number of tandem repeats (VNTR) analysis (MLVA) system [8], [9], but in an evaluation it was found that some VNTR markers may vary with replication of single clones and cause difficulties in interpretation [10]. In our study, we used the MLST system developed by Klint et al. for *C. trachomatis* based on PCR amplification and DNA sequencing of five highly variable target regions (not house keeping genes), that has displayed a three-fold higher resolution than *ompA* sequencing [11] and with a resolution similar to MLVA [12]. The target stability of this MLST scheme has proved satisfactory through sequencing studies of the nvCT [3], [13], [14] and of lymphogranuloma venereum *C. trachomatis* strains [15]. The scheme has been applied in several Swedish studies [11], [13], and the multilocus sequence types (STs) have been included in the Uppsala University *C. trachomatis* MLST database (http://mlstdb.bmc.uu.se) enabling us to compare STs sampled in our study to STs collected in Sweden. We expected to find a proportion of common *C. trachomatis* STs in neighbouring countries Norway and Sweden, including the nvCT.

The aims of our study were: i) to examine distribution of *C. trachomatis* STs in an adolescent population in an unmapped high-incidence area in North Norway, ii) to compare the genetic diversity in a remote sparsely populated county with that of two urban regions in Norway, and iii) to compare the discriminatory capacity of the MLST scheme developed by Klint et al. with conventional *ompA* sequencing by applying both methods to a large number of chlamydia specimens from different geographic locations. To achieve this, we conducted a population based cross-sectional study collecting chlamydia specimens from high school students in Finnmark county, an extended county with minor municipalities and a population of only 72,500 (www.ssb.no, Statistics Norway). Additional chlamydia specimens from adolescent girls and boys were collected from routine clinical samples in Tromsø and Trondheim, capitals of North and Central Norway, respectively. Our approach resulted in a total of 248 *C. trachomatis* specimens that were successfully genotyped, enabling us to assess genetic diversity within the different catchment areas, and compare the resolution of the two methods.

## Methods

### Study population and urine sampling

A population based cross-sectional study was conducted among girls and boys in five senior high schools in Finnmark county during fall 2009 (manuscript in preparation). Briefly, the participants filled in a web-based questionnaire on demography, sexual behaviour and urogenital symptoms, and provided first-void urine samples under supervision of the study staff, giving a total of 60 chlamydia specimens from 1,476 urine samples that were analysed at the laboratory at the University Hospital of North Norway (UNN, Tromsø). Parallel to the high school study, 20 and 80 chlamydia positive urine samples from 15–20 year-olds in Finnmark and the Tromsø region, respectively, were consecutively collected from routine clinical samples at UNN Tromsø. Eighty-eight samples from patients of the same age group in the Trondheim region were collected at St. Olavs Hospital (Central Norway). After processing, a total of 248 chlamydia samples were immediately frozen at −70°C in the laboratories and later transported on dry ice to the University Hospital of Uppsala (Sweden) for genotyping.

### Laboratory testing of urine samples

#### Chlamydia PCR

The UNN laboratory extracted DNA using the BUGS'n BEADS TM-STI kit (NorDiag ASA, Oslo, Norway) and used the ProCt real-time PCR (ProCelo A/S, Tromsø, Norway) with sensitivity 97% and specificity 100%. The Trondheim laboratory prepared DNA using the bacterial protocol on GenoM 48 (Qiagen, Hilden, Germany) and used an in-house triplex real-time PCR (cryptic plasmid, MOMP gene and internal control) with sensitivity 96% and specificity 100% [16]. A plasmid specific PCR was used to confirm MLST identification of the nvCT [17].

#### Strain typing

*ompA* sequence determination was performed according to a previously described method [18] and strains were categorized into genovars D–K and *ompA* genotypes. Genovars denote subgroups of *C. trachomatis* based on serospecificity for MOMP inferred from *ompA* sequencing. Genotypes are subgroups based on *ompA* sequencing. The MLST scheme comprises five highly variable target regions and was performed as previously described [11] except that the *pbpB* region was amplified as two separate fragments according to Jurstrand et al. [13]. Allele numbers were assigned by comparing the sequence at each locus to all known corresponding alleles available in the Uppsala University *C. trachomatis* MLST database (http://mlstdb.bmc.uu.se). Allele profiles based on the five genetic regions are expressed as multilocus sequence types (STs). At baseline date February 16^th^ 2010, the database included 145 STs originating from 467 chlamydia isolates. In our study, clonal complexes are defined as clusters of genetically related STs with only one allele difference, i.e. single-locus variants (SLVs). The founder of a clonal complex is the ST that differs from the largest number of other STs at only a single locus, i.e. the ST that has the highest number of SLVs.

### Ethics

In the high school study, written informed consent was obtained from the next of kin, carers or guardians on the behalf of participants younger than 16 years. Participants 16 years or older gave their informed consent by filling in a web-based questionnaire in accordance with the Health Research Act §17.b stating their right to consent. All procedures were approved by the Regional Committees for Medical and Health Research Ethics North Norway (REK Nord No.: 200900528-6/MRO/400) and the Data Protection Officer at UNN (No.: 2009/2475). Establishment of a research bio-bank for *C*. *trachomatis* specimens was approved by The National Directorate of Health (Bio-bank Registry No. 2723).

### Statistical methods

SPSS 18.0 for Windows was used for statistical analysis of the associations between urogenital symptoms, and STs and clonal complexes (chi-square). Binominal confidence intervals were calculated according to Clopper-Pearsson exact method [19]. The discriminatory power (*D*) of a typing method refers to the probability that two unrelated strains sampled from the test population will be placed into different typing groups. *D* was determined for *ompA* genotyping and MLST in the 188 routine clinical samples using Hunter and Gaston's modification of Simpson's discriminatory index [20]:
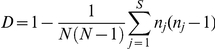
where *N* is the number of unrelated strains tested, *s* is the number of different types, and *n_j_* is the number of strains belonging to the *j*th type. Confidence interval (CI) for *D* was calculated as originally described by Simpson [21]. A cut-off value for *D* of ≥0.95 for a molecular typing method is considered ‘ideal’ [22]. As the 188 samples were consecutively collected in the laboratories from a defined age group and within a limited time frame from defined geographic areas, a degree of epidemiological relatedness could not be excluded. The following assumptions were made: the two most common STs in an ‘ideal’ epidemiologically independent sample will have prevalences equal to the third most prevalent ST. Thus, *n_ST12_* and *n_ST56_* were set equal to ST153 (n = 15), and a corrected *D_c_* was calculated. BioNumerics software (version 6.01, Applied Maths, Sint-Martens-Latem, Belgium) was used to generate a minimum spanning tree under the categorical coefficient of similarity and the priority rule of the highest number of single-locus variants.

## Results

A complete MLST profile was obtained for all 248 chlamydia specimens identifying a total of 50 STs (Table 1). *ompA* sequencing detected 11 genotypes, thus the MLST scheme provided 4.5 higher resolution than *ompA*. By combining MLST and *ompA*, 53 unique genotypes were identified. The commonly predominating *ompA* E/Bour genotype comprised 46% of all specimens and could be further resolved by the MLST system into 24 different STs, i.e. giving 24 times higher resolution. Nineteen percent of all specimens belonged to genovar G which could be further resolved into nine different STs.

**Table 1:** Number of genetic variants of *ompA* sequencing and multilocus sequence typing (MLST) within each genovar (D–K) in 248 *C. trachomatis* specimens.

Chlamydia genovars			Number of genetic variants		
Genovar	No of specimens	(%)	*ompA* genotypes	STs^1^	STs with *ompA*
D	21	(8.5)	2	3	3
E	115	(46.4)	2	24	25
F	44	(17.7)	1	6	6
G	47	(19.0)	2	9	9
H	5	(2.0)	1	2	2
I	1	(0.4)	1	1	1
J	1	(0.4)	1	1	1
K	14	(5.6)	1	6	6
Total	248	(100.0)	11	50^2^	53^2^

1 Sequence types (STs) of *C. trachomatis* detected by MLST.

2 The numbers reflect the total number of unique genetic variants in the 248 chlamydia specimens and do not equal the sum of each column.

Simpson's discriminatory index (*D*) was calculated in the 188 routine clinical samples (Table S1) and was 0.93 (95% CI 0.91–0.95) for MLST and 0.67 (95% CI 0.61–0.73) for *ompA* sequencing, respectively. A corrected *D*_c_ of 0.97 (95% CI 0.96–0.98) was calculated for MLST.

Among the 50 STs, 31 STs (62%) were novel, while 19 STs had been identified previously. Novel STs were numbered in order of identification: ST146–ST176 (Table S2). Four of the 50 STs were singletons, i.e. differing at more than two alleles from all other isolates. Fifty-two percent of the STs comprised only one specimen and 62% had less than four specimens.

A total of 12 new alleles in the MLST scheme were detected comprising 9% of all specimens (Table S2). The three most variable regions, *pbpB*, *hctB* and CT058 displayed five, three and three new alleles, respectively. The less variable regions CT144 had one new allele and CT172 had none. Most of the new alleles were substitutions of a single base pair.

**Figure 1:**
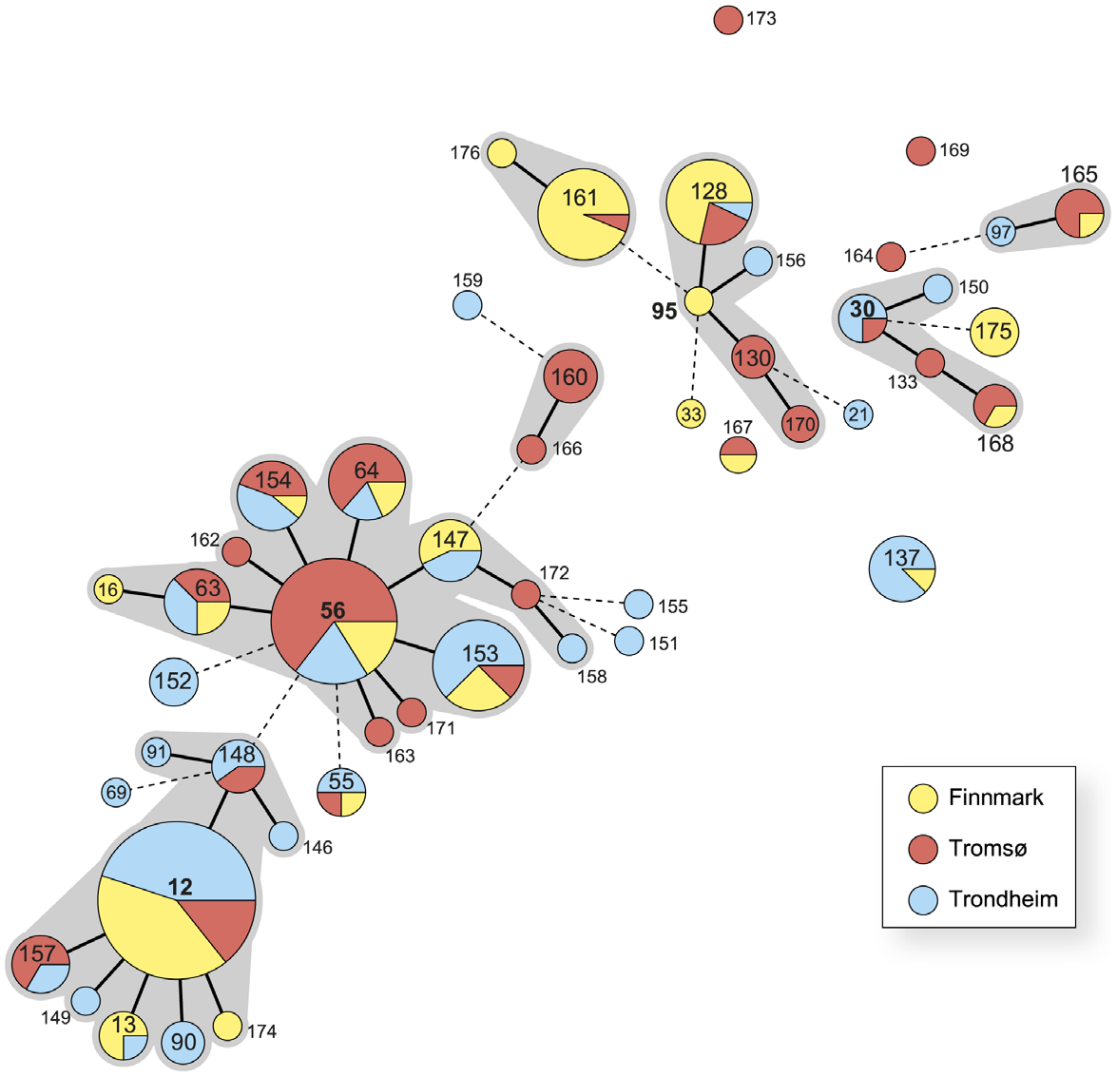
Minimum spanning tree analysis based on 50 multilocus sequence types (STs) in 248 chlamydia specimens. The 50 circles correspond to different STs discriminated by multilocus sequence typing (MLST). Each circle represents an ST, and ST number is given inside or next to the circle. Circle size reflects the number of isolates. Bold black lines connect single-locus variants (SLV). Broken lines connecting double-locus variants are only indicative as several alternative links with equal weight may exist. The coloured pie charts indicate ST geographic distribution. Grey shaded areas define clonal complexes i.e. clusters of genetically related STs with only one allele difference.

All 248 chlamydia specimens were clustered using a minimum spanning tree based on the STs (Figure 1). ST12, ST30, ST56, and ST95 were considered putative founders of a clonal complex. All four were present in the MLST database prior to our study. ST12 (20%) and ST56 (13%) were also the most frequent clones and were present at all three collection sites. Of all specimens, 57% (142 of 248) belonged to STs present in all three areas, and included eight STs, of which ST153 and ST154 were new. Sixty-four percent (32 of 50) of the STs were unique for specific areas. Differences in genetic diversity as estimated by ST variation and proportion of novel STs were not statistically significant between the three geographic areas (Table 2).

**Table 2:** Geographic distribution of *C. trachomatis* specimens according to three different strain typing methods, and genetic diversity within each area.

Geographic area	Finnmark^1^	Tromsø	Trondheim	Total^2^
No of chlamydia specimens	80	80	88	248
No of STs^3^	21	27	28	50
No of novel STs	10	18	14	31
No of cases with novel STs	33	34	34	101
ST variation^4^ (95% CI)	0.26 (0.17–0.40)	0.34 (0.23–0.44)	0.32 (0.22–0.41)	0.20 (0.15–0.25)
% novel STs^5^ (95% CI)	47.6 (26.3–69.0)	66.7 (48.9–84.5)	50.0 (31.5–68.5)	62.0 (48.6–75.5)
No of genovars^6^ D–K	6	7	7	8
No of *ompA* genotypes^7^	7	10	9	11

CI: Confidence interval.

1 *C. trachomatis* specimens from either the high school study (n = 60) or routine clinical samples in the laboratory (n = 20).

2 The numbers reflect the results for all 248 specimens and do not necessarily equal the sum of each row.

3 Sequence types of *C. trachomatis* detected by multilocus sequence typing.

4 Number of STs identified in an area divided by number of chlamydia specimens in the area.

5 Percentage novel STs in an area of total number of STs in the area.

6 Genovar D–K of *C. trachomatis* inferred from *ompA* sequencing.

7 Genotypes of *C. trachomatis* detected by *ompA* sequencing.

Four of the 248 specimens were identified as ST55 which appears to be unique to the new Swedish variant of *C. trachomatis* (nvCT) [17]. One nvCT specimen was found in Finnmark and Tromsø, respectively, as were two in Trondheim.

Genovar E was most prevalent in both Tromsø (65%, 95% CI 54–75%) and Trondheim (48%, 95% CI 37–59%) and significantly more frequent than in Finnmark (26%, 95% CI 17–36%). Finnmark had the most atypical genovar distribution with G being predominant, contributing 36% (95% CI 26–47%), which was significantly higher than in Tromsø (13%, 95% CI 6–22%) and Trondheim (9%, 95% CI 4–17%). The predominance of genovar G in Finnmark was mainly due to the expansion of ST128 and the novel ST161 (Figure 1).

In Finnmark, ST12 (25%), ST161 (19%) and ST128 (13%) were the most prevalent chlamydia clones (Figure 1). The novel ST161 was almost unique for Finnmark. It was identified in four of the five high schools and was equally distributed between the genders. In subjects infected with ST161, there were as many symptomatic as asymptomatic individuals. ST128 was not prevalent in Tromsø (3%) or Trondheim (1%).

Among the 20 STs identified in the Finnmark high school study, six STs were found in both genders, twelve STs were present in girls only, and two STs were present only in boys. The founders ST12 and ST56, and the novel ST161, were among the six STs shared between genders. Among the two STs found in boys only, one specimen of ST33 was identified in a male participant in Finnmark who reported having sex with men. Chlamydia infected girls had a higher proportion of samples with gender-specific STs (34%, 95% CI 20–51%) compared to infected boys (11%, 95% CI 1.3–33%).

Among participants in the high school study, 59% of chlamydia infected girls and 22% of infected boys (*p = *0.01) reported urogenital symptoms. No statistically significant associations between clinical symptoms and specific STs or clonal complexes were found.

## Discussion

This is the largest study to date using this MLST system and is also the study where MLST has outperformed *ompA* the most by offering a five-fold higher resolution than *ompA* genotyping, compared with the three-fold increase described earlier [11]. We observed a discriminatory index *D* of 0.93 (95% CI 0.91–0.95) which was slightly lower than expected in such a large number of samples. A cut-off value*≥*0.95 is considered ‘ideal’ for molecular typing methods [22]. The high prevalence of ST12 and ST56 could indicate that the 188 laboratory samples were not completely epidemiologically independent, and we therefore decided to use a prevalence correction for the two most frequent STs. We calculated a significantly higher corrected *D_c_* of 0.97 (95% CI 0.96–0.98) with the entire confidence interval above the cut-off value. Two previous studies fulfilling the above sampling criteria, but with only a small number of samples (both n = 31) reported *D* between 0.95 and 0.96 for this MLST scheme [10], [12]. Confidence intervals were not assessed in these studies. As *D* includes no correcting factor for small populations, typing schemes should not be validated with small samples [20].

The MLST scheme resolved the chlamydia specimens into a number of STs of which a significant proportion comprised only a few specimens and two-thirds were novel. The minimum spanning tree analysis (Figure 1) showed that the majority of specimens belonged to clonal complexes which have also been observed in other bacterial MLST databases [23]. Organization into clonal complexes makes MLST data more suitable to epidemiologic analysis and reduces the potential of over-discrimination. The multiple novel STs could be due to the relatively short existence of the database only since 2007. In addition, genotyping of chlamydia strains from individuals in an unmapped geographic area will commonly identify a number of novel STs. As the database expands with time, it is expected that genetic relationships between more STs will be revealed. Prior to this study, Norwegian chlamydia specimens from heterosexuals had not been characterized using this MLST scheme.

Among the founders of clonal complexes, ST12 was the most prevalent constituting one-fifth of the strains in all three areas. ST12 is common among both heterosexuals and men having sex with men (MSM) in Sweden and other European countries. ST30 and ST56 are also frequently reported to the database. The founder ST95 (one female, Finnmark) had previously been identified in only three samples from Dutch females illustrating how an individual through sexual contact might have interconnected geographically distant areas.

A nvCT prevalence of 1.6% was as expected as nvCT has rarely been identified outside Sweden [24]. These infections could have been imported directly from Sweden, but may also reflect domestic spread. As the questionnaire did not include ethnicity or origin country of former sex partners, we could not examine any links to Sweden. A previous study found that the nvCT prevalence in Oslo increased from 1.0% in the first quarter of 2007 to 3.4% in the second quarter of 2008, indicating a slow spread within Norway [25]. The laboratories in Tromsø and Trondheim have used nvCT sensitive diagnostic assays since 2005 and 2006, respectively, implying that the nvCT clone has not escaped detection in these areas.

One specimen from Finnmark contained an ST33 genotype which had previously only been found among MSM in Stockholm (Sweden) and France. ST33 was detected in a Finnmark male who reported having sex with men which could indicate links to international MSM networks. Due to limited epidemiological data on previous sex partners we could not confirm this hypothesis. The discrimination of nvCT and ST33 is not possible using *ompA* sequencing.

Genovar E was the most common genovar in Tromsø and Trondheim, as in heterosexual populations elsewhere [5], [26]. The predominance of genovar G in Finnmark is unusual in heterosexual populations and was mainly due to the expansion of ST128 and ST161. As the 20 routine clinical samples also were restricted to the 15–20 year-olds, we could not determine whether the genovar distribution in our study reflects the distribution in the general population in Finnmark. The uniquely high occurrence of ST128 and ST161 in Finnmark and no significant spread to neighbouring Tromsø may be explained by these clones being limited to local sexual networks in Finnmark. However, we could not confirm this hypothesis due to lack of sexual network information.

Possible factors contributing to the success of ST12, ST128 and ST161 in Finnmark could be a high transmission rate reflecting increased tissue tropism, or the strains causing a silent infection escaping discovery. However, all three strains were symptomatic in approximately half of infected participants in the high school study. Due to only 60 chlamydia specimens carrying behavioural data, the reasons for the success of ST12, ST128 and ST161 cannot be further elucidated. As previously shown, no associations between urogenital symptoms and specific STs or clonal complexes were found [27].

The chlamydia infected girls in the high school study had a higher proportion of gender-specific STs compared to boys. This may indicate that a significant proportion of female students had off-school sex partners, and therefore were infected with STs not identified in their high school male peers. This was supported by the girls reporting older partners at last intercourse (19.9 years) compared to the boys (16.3 years, *p*<0.01). The propensity of young girls to have older partners has also been shown in other studies [28].

The achievement of a complete MLST profile for all 248 samples was unexpected compared to previous studies. However, all specimens were new and fresh, they were frozen at −70°C immediately after the first diagnostic PCR, and they were thawed for the first time prior to MLST to avoid degradation of DNA. In addition, the MLST method has been optimized since the introduction in 2007 which also could have contributed to the high success rate [13]. Thus, we consider the results reliable.

Presently our MLST system is too labour intensive to enable epidemiological analysis in clinical routine with partner notification. Future research should focus on development of a typing scheme with a high discriminatory power that allows for rapid and easy interpretation, but which also is economically affordable. Next generation sequencing technologies may in the future reach this objective. In an area where the chlamydia STs are known, array-based methods for analysis of sequence variation might be an alternative, but this approach will not detect STs with novel alleles [29].

In conclusion, our study shows that this MLST scheme is a valuable tool for studying the molecular epidemiology of *C. trachomatis* infections and far superior to *ompA* typing in terms of resolution especially of the globally predominant genovar E.

## Supporting Information

Table S1 **188** ***C. trachomatis*** **specimens from routine clinical samples in the laboratories resolving into 46 multilocus sequence types (STs) listed by: ST number, the corresponding** ***ompA*** **genotype and genovar (D–K), and number of specimens within each ST.**
(DOC)

Table S2 **248** ***C. trachomatis*** **specimens resolving into 50 multilocus sequence types (STs) listed by: ST number, the five specific alleles making up the MLST profile, the corresponding** ***ompA*** **genotype and genovar (D–K), and number of specimens within each ST.**
(DOC)
