# Supplementary material for: Multilocus Sequence Typing of Genital Chlamydia trachomatis in Norway Reveals Multiple New Sequence Types and a Large Genetic Diversity
Source: PLoS One. 2012 Mar 28;7(3):e34452. doi: 10.1371/journal.pone.0034452 (PMC3314642; doi:10.1371/journal.pone.0034452)
Supplement: Table S1 — 188 C. trachomatis specimens from routine clinical samples in the laboratories resolving into 46 multilocus sequence types (STs) listed by: ST number, the corresponding ompA genotype and genovar (D–K), and number of specimens within each ST. (DOC) [file pone.0034452.s001.doc]

Table S1. 188 *C. trachomatis* specimens from routine clinical samples in the laboratories, resolving into 46 multilocus sequence types (STs) listed by: ST number, the corresponding *ompA* genotype and genovar type (D-K), and number of specimens within each ST.

| **ST** | ***ompA* genotype** | **Genovar D-K** | **No of specim.** |
| --- | --- | --- | --- |
| 12 | 1, 24, 6 | D, F, E | 35 |
| 13 | 24 | F | 2 |
| 21 | 2 | D | 1 |
| 30 | 12 | K | 4 |
| 55^1^ | 6 | E | 3 |
| 56 | 6 | E | 28 |
| 63 | 6 | E | 6 |
| 64 | 6, 7 | E | 10 |
| 69 | 6 | E | 1 |
| 90 | 24 | F | 3 |
| 91 | 24 | F | 1 |
| 95 | 11 | G | 1 |
| 97 | 35 | H | 1 |
| 128 | 11, 41 | G | 5 |
| 130 | 11 | G | 3 |
| 133 | 12 | K | 1 |
| 137 | 11 | G | 7 |
| 146 | 6 | E | 1 |
| 147 | 6 | E | 5 |
| 148 | 24 | F | 5 |
| 149 | 24 | F | 1 |
| 150 | 12 | K | 1 |
| 151 | 6 | E | 1 |
| 152 | 6 | E | 4 |
| 153 | 6 | E | 15 |
| 154 | 6 | E | 8 |
| 155 | 6 | E | 1 |
| 156 | 36 | I | 1 |
| 157 | 6 | E | 6 |
| 158 | 6 | E | 1 |
| 159 | 6 | E | 1 |
| 160 | 6 | E | 5 |
| 161 | 11 | G | 3 |
| 162 | 6 | E | 1 |
| 163 | 6 | E | 1 |
| 164 | 11 | G | 1 |
| 165 | 35 | H | 3 |
| 166 | 6 | E | 1 |
| 167 | 2 | D | 1 |
| 168 | 12 | K | 2 |
| 169 | 20 | J | 1 |
| 170 | 11 | G | 2 |
| 171 | 6 | E | 1 |
| 172 | 6 | E | 1 |
| 173 | 12 | K | 1 |
| 175 | 12 | K | 1 |
| n=46 | n=11 | n=8 | n=188 |

^1^new Swedish mutant of *C. trachomatis*
