# Supplementary material for: Multilocus Sequence Typing of Genital Chlamydia trachomatis in Norway Reveals Multiple New Sequence Types and a Large Genetic Diversity
Source: PLoS One. 2012 Mar 28;7(3):e34452. doi: 10.1371/journal.pone.0034452 (PMC3314642; doi:10.1371/journal.pone.0034452)
Supplement: Table S2 — 248 C. trachomatis specimens resolving into 50 multilocus sequence types (STs) listed by: ST number, the five specific alleles making up the MLST profile, the corresponding ompA genotype and genovar (D–K), and number of specimens within each ST. (DOC) [file pone.0034452.s002.doc]

Table S2. 248 *C.trachomatis* specimens resolving into 50 multilocus sequence types (STs) listed by: ST number, the five specific alleles making up the MLST profile, the corresponding *ompA* genotype and genovar (D-K), and number of specimens within each ST.

|  | **MLST profile** | | | | |  |  |  |
| --- | --- | --- | --- | --- | --- | --- | --- | --- |
| **ST** | ***hctB*** | **CT058** | **CT144** | **CT172** | ***pbpB*** | ***ompA* genotype** | **Genovar D-K** | **No of specim.** |
| 12 | 5 | 19 | 7 | 1 | 4 | 1, 24, 6 | D, F, E | 49 |
| 13 | 5 | 19 | 15 | 1 | 4 | 24 | F | 4 |
| 16 | 7 | 19 | 14 | 2 | 1 | 6 | E | 1 |
| 21 | 10 | 4 | 1 | 4 | 23 | 2 | D | 1 |
| 30 | 10 | 7 | 9 | 3 | 8 | 12 | K | 4 |
| 33 | 10 | 8 | 5 | 3 | 6 | 11 | G | 1 |
| 55¹ | 21 | 19 | 1 | 2 | 1 | 6 | E | 4 |
| 56 | 1 | 19 | 7 | 2 | 1 | 6 | E | 31 |
| 63 | 7 | 19 | 7 | 2 | 1 | 6 | E | 8 |
| 64 | 4 | 19 | 7 | 2 | 1 | 6, 7 | E | 11 |
| 69 | 5 | 19 | 6 | 2 | 2 | 6 | E | 1 |
| 90 | 5 | 19 | 1 | 1 | 4 | 24 | F | 3 |
| 91 | 5 | 19 | 5 | 2 | 4 | 24 | F | 1 |
| 95 | 10 | 8 | 1 | 3 | 5 | 11 | G | 1 |
| 97 | 12 | 12 | 11 | 9 | 21 | 35 | H | 1 |
| 128 | 10 | 8 | 1 | 4 | 5 | 11, 41 | G | 14 |
| 130 | 10 | 4 | 1 | 3 | 5 | 11 | G | 3 |
| 133 | 10 | 7 | 9 | 1 | 8 | 12 | K | 1 |
| 137 | 10 | 36 | 22 | 4 | 6 | 11 | G | 8 |
| 146 | 5 | 19 | 7 | 2 | 47* | 6 | E | 1 |
| 147 | 1 | 19 | 7 | 2 | 2 | 6 | E | 7 |
| 148 | 5 | 19 | 7 | 2 | 4 | 24 | F | 5 |
| 149 | 5 | 19 | 7 | 1 | 48* | 24 | F | 1 |
| 150 | 10 | 7 | 9 | 3 | 49* | 12 | K | 1 |
| 151 | 1 | 2 | 6 | 2 | 50* | 6 | E | 1 |
| 152 | 47* | 38* | 7 | 2 | 1 | 6 | E | 4 |
| 153 | 35 | 19 | 7 | 2 | 1 | 6 | E | 16 |
| 154 | 48* | 19 | 7 | 2 | 1 | 6 | E | 9 |
| 155 | 12 | 2 | 6 | 2 | 2 | 6 | E | 1 |
| 156 | 10 | 8 | 1 | 3 | 18 | 36 | I | 1 |
| 157 | 5 | 19 | 7 | 1 | 1 | 6 | E | 6 |
| 158 | 5 | 2 | 7 | 2 | 2 | 6 | E | 1 |
| 159 | 35 | 2 | 1 | 1 | 2 | 6 | E | 1 |
| 160 | 10 | 2 | 1 | 2 | 2 | 6 | E | 5 |
| 161 | 8 | 5 | 1 | 3 | 5 | 11 | G | 16 |
| 162 | 1 | 19 | 7 | 2 | 51* | 6 | E | 1 |
| 163 | 1 | 39* | 7 | 2 | 1 | 6 | E | 1 |
| 164 | 12 | 12 | 25* | 9 | 5 | 11 | G | 1 |
| 165 | 12 | 12 | 9 | 9 | 21 | 35 | H | 4 |
| 166 | 10 | 19 | 1 | 2 | 2 | 6 | E | 1 |
| 167 | 12 | 15 | 1 | 3 | 23 | 2 | D | 2 |
| 168 | 10 | 7 | 9 | 1 | 13 | 12 | K | 3 |
| 169 | 29 | 5 | 12 | 4 | 18 | 20 | J | 1 |
| 170 | 10 | 4 | 1 | 3 | 6 | 11 | G | 2 |
| 171 | 49* | 19 | 7 | 2 | 1 | 6 | E | 1 |
| 172 | 1 | 2 | 7 | 2 | 2 | 6 | E | 1 |
| 173 | 10 | 40* | 16 | 4 | 21 | 12 | K | 1 |
| 174 | 5 | 19 | 7 | 1 | 2 | 6 | E | 1 |
| 175 | 10 | 6 | 22 | 3 | 8 | 12 | K | 4 |
| 176 | 8 | 5 | 5 | 3 | 5 | 11 | G | 1 |
| n=50 | n=13 | n=13 | n=12 | n=5 | n=15 | n=11 | n=8 | n=248 |

*novel allele, ¹new Swedish mutant of *C. trachomatis*
